# Retraction: Implementation of organization and end-user computing-anti-money laundering monitoring and analysis system security control

**DOI:** 10.1371/journal.pone.0320467

**Published:** 2025-03-18

**Authors:** The PLOS One Editors

## Introduction

The *PLOS One* Editors retract this article [1] because it was identified as one of a series of submissions for which we have concerns about peer review integrity and potential manipulation of the publication process. These concerns call into question the validity and provenance of the reported results. We regret that the issues were not identified prior to the article’s publication.

The author either did not respond directly or could not be reached.
